# Obstructive sleep apnea and CPAP efficacy in patients with Hypermobile Ehlers-Danlos syndrome and hypermobility spectrum disorder: a case-control study

**DOI:** 10.1007/s11325-026-03763-3

**Published:** 2026-07-08

**Authors:** Kenneth L. Zhang, Rebecca Wallon, Christina M. Laukaitis, Charles Davies

**Affiliations:** 1 Carle Illinois College of Medicine, 506 S Mathews Ave, Urbana, IL 61801 USA; 2 Carle Health, Urbana, IL USA; 3 Carl R. Woese Institute for Genomic Biology, Urbana, IL USA

**Keywords:** Hypermobile Ehlers-Danlos Syndrome, Hypermobility Spectrum Disorder, Obstructive sleep apnea, Case-control study, CPAP

## Abstract

**Purpose:**

Hypermobile Ehlers-Danlos syndrome (hEDS) and Hypermobility Spectrum Disorder (HSD) are hereditary tissue disorders existing on a spectrum associated with insomnia and obstructive sleep apnea (OSA). Oropharyngeal abnormalities in hEDS/HSD may exacerbate OSA; this study investigates if continuous positive airway pressure (CPAP) adequately decreases daytime sleepiness in hEDS/HSD patients with OSA.

**Methods:**

A single institution retrospective review was conducted from 2020 to 2024. Adults with hEDS/HSD and OSA (cases) were matched based on sex, age, race, and body mass index (BMI) to adults with OSA (controls). Data from polysomnogram (PSG), sleep clinic consults, and reevaluation 3–9 months post-CPAP were analyzed. The Epworth Sleepiness Scale (ESS) was used to assess daytime sleepiness.

**Results:**

68 cases (47% hEDS/53% HSD, Beighton score 5.71 ± 2.29, age 34.07 ± 13.28, BMI 32.88 ± 8.47) were matched to 68 controls. All pairs completed PSG; 53 pairs pursued CPAP; 41 pairs were reevaluated. Cases had lower sleep efficiency (77%±16% vs. 82%±10%, *p* < 0.05) and higher insomnia prevalence (44.7% vs. 34.3%, *p* < 0.05). Pre-CPAP ESS was similar (10.39 ± 4.89 vs. 11.22 ± 5.12, *p* > 0.05). CPAP reduced ESS in controls (− 4.46, *p* < 0.05) but not in cases (− 1.56, *p* > 0.05). Cases and controls had similar CPAP compliance (75.6%±25.2% vs. 74.6%±26.7%, *p* > 0.05), Apnea-Hypopnea Index (AHI) (7.2 [13.6] vs. 7.0 [12.7], *p* > 0.05), lowest SpO_2_ (86.08%**±**4.31% vs. 85.67%**±**3.52%, *p* > 0.05), and residual AHI (1.55 [1.75] vs. 1.2 [1.35], *p* > 0.05). Cases used nasal masks less frequently (22.6% vs. 41.5%, *p* < 0.05).

**Conclusion:**

CPAP was not associated with a statistically significant improvement in daytime sleepiness in hEDS/HSD patients despite effective treatment, suggesting that hypermobility-related factors beyond OSA may contribute to residual sleepiness.

## Introduction

Hypermobile Ehlers–Danlos syndrome (hEDS), the most common subtype of Ehlers–Danlos syndrome (EDS), is a hereditary connective tissue disorder with skin hyperextensibility, joint hypermobility, and tissue fragility. Hypermobility spectrum disorder (HSD) describes individuals with joint hypermobility who do not meet full diagnostic criteria for hEDS [1]. Both conditions share a range of extra-articular manifestations, and the substantial overlap in features and sequelae suggests hEDS and HSD exist along a continuum rather than as distinct disorders [2]. Collectively, patients with hEDS/HSD will be referred to as hypermobile patients.

Individuals with EDS are at higher risk for obstructive sleep apnea (OSA) [3]. A meta-analysis reported that hypermobile patients are six times more likely to develop OSA [4] and a small exploratory study found sleep disordered breathing in all EDS patients evaluated with polysomnogram (PSG) [5]. This elevated risk is thought to arise from the connective tissue abnormalities that characterize hEDS. Defective collagen synthesis and connective tissue fragility may increase laxity in their pharyngeal walls, soft palate, and laryngeal structures [6]. In addition, cartilaginous defects and altered craniofacial development, both commonly reported in hEDS, may further narrow the airway [7]. Dental crowding accompanied by a high or narrow palate are particularly prevalent in the hEDS population and is recognized as one of twelve diagnostic systemic manifestation of the disorder [1]. Together, tissue laxity and skeletal abnormalities reduces upper airway support, making it prone to collapse during sleep when muscle tone decreases [6, 7]. Although these mechanisms may increase the likelihood of OSA in hypermobile patients, their impact on OSA severity independent of BMI has not been studied.

The effectiveness of continuous positive airway pressure (CPAP) in improving daytime sleepiness in hypermobile patients also remains poorly understood. Common extra-articular comorbidities in hypermobile patients include autonomic dysfunction, insomnia, anxiety, and depression, which may independently influence sleep and limit improvements in sleepiness with CPAP [8]. OSA in hypermobile individuals is associated with greater fatigue, excessive daytime sleepiness, and reduced quality of life when compared with controls matched on sex, age, weight, and height [3]. Given that 77% of EDS patients suffer from severe fatigue [9], the interplay between chronic fatigue and sleep quality may worsen daytime sleepiness and hinder CPAP effectiveness [10, 11].

The primary aim of this study is to compare the effect of CPAP on daytime sleepiness between hypermobile patients with OSA and matched controls in a one-to-one case–control design. Secondary objectives include assessing differences in PSG metrics, CPAP usage, OSA severity, and insomnia comorbidity between hypermobile patients and matched controls.

## Methods

### Participant Selection

A retrospective case–control study was conducted of patients diagnosed with OSA between 2020 and 2024 at a single institution. This study was approved by the Carle Foundation Hospital Institutional Review Board (project No. 24NPR3999). Only patients who completed an in-clinic PSG were eligible. OSA was defined as an apnea–hypopnea index (AHI) ≥ 5 events/hour; when AHI was unavailable, respiratory disturbance index (RDI) was used. No power calculation was performed for a specific effect size due to the exploratory nature of this study. The sample size was based on available cases meeting inclusion criteria. Results should therefore be interpreted as hypothesis-generating rather than confirmatory.

Cases were defined as patients with OSA and a comorbid diagnosis of hEDS or HSD (ICD-10 codes Q79.6, Q79.62, M35.7). To minimize misclassification, all hEDS/HSD diagnoses were reviewed and confirmed based on documented clinical evaluations and the 2017 International Classification of EDS diagnostic framework [1]. Each case was matched 1:1 to a control patient with OSA without a hypermobility-related disorder based on sex, age, race, and BMI recorded at the diagnostic PSG. Exclusion criteria for cases and controls included age < 18 years; chronic opioid or benzodiazepine use; high-dose stimulant use; and comorbidity of bipolar disorder, schizophrenia, rapid eye movement sleep behavior disorder, severe chronic obstructive pulmonary disease, or heart failure with reduced ejection fraction. We did not exclude patients using common sedating or activating medications such as non-opioid analgesics, antidepressants, antihistamines, or prescribed sleep aids given their high prevalence in real-world patients with OSA and hEDS/HSD. Exclusion would limit generalizability of this study.

### PAP therapy protocol

After diagnostic PSG, patients pursued either (1) initiation of auto-titrating PAP (APAP) with standard initial pressure range of 4–20 cm H₂O, or (2) completion of an in-laboratory PAP calibration study followed by fixed-pressure CPAP prescription. Patients met with a respiratory therapist at their durable medical equipment provider for CPAP setup and education. Mask fitting was performed in person trialing multiple interfaces to optimize comfort, seal, and tolerance. Final mask selection was patient driven. Chin straps to address oral leak was discussed. During follow-up visits, providers reviewed objective CPAP data, assessed patient-reported comfort issues, and individualized adjustments.

### Data extraction

After matching, data were manually extracted from electronic medical record into predefined fields in REDCap [12] by a trained reviewer. Periodic quality checks ensured data accuracy. Data sources included initial Sleep Medicine consultations, diagnostic PSG, 3–9-month follow-up visits after CPAP initiation, and initial consultations for hEDS/HSD diagnosis. Categories of collected data included demographics, hypermobility, PSG, CPAP, and sleepiness. The Epworth Sleepiness Scale (ESS) was used to assess daytime sleepiness [13]. Patient perspectives regarding CPAP side effects mentioned during follow-up visits were extracted and thematic analysis was conducted. Patients with missing data for specific variables were retained for sub-analyses; missing data were handled by pairwise deletion. For post-CPAP analyses, only matched pairs with both returning for follow-up were included. Because analyses relied on pairwise deletion at follow-up, effective sample sizes varied across outcomes and estimates assume that missingness was random. Violations of this assumption may introduce selection bias. Additionally, no further adjustments were performed for variables that remained imbalanced after matching, such as presence of insomnia, OSA severity, or baseline sleepiness. Therefore, residual confounding cannot be excluded.

### Statistical analysis

Following data collection, a pseudonymized dataset was exported for analysis. ESS was compared pre and post CPAP for both groups. PSG and CPAP metrics were compared between groups. A subgroup analysis compared cases with dental crowding and a high-arched palate to their matched controls based on the known association between craniofacial abnormalities and OSA severity. The Beighton score is a nine-point clinical scale used to assess generalized joint hypermobility with one point assigned per designated joint displaying hypermobility [14]. The Beighton score was used as a surrogate for hypermobility severity to correlate with OSA severity. Categorical variables were compared using McNemar or Stuart–Maxwell tests. Continuous variables were compared using paired t-tests. Spearman’s rho was used for nonparametric correlational analyses between ordinal and continuous variables. AHI was presented as median [IQR] due to non-normal distribution and is compared between groups using a Mann–Whitney U test. Data were cleaned in Microsoft Excel, then analyzed and graphed using GraphPad Prism (version 10.6.1, GraphPad Software, San Diego, CA).

## Results

**Fig. 1:**
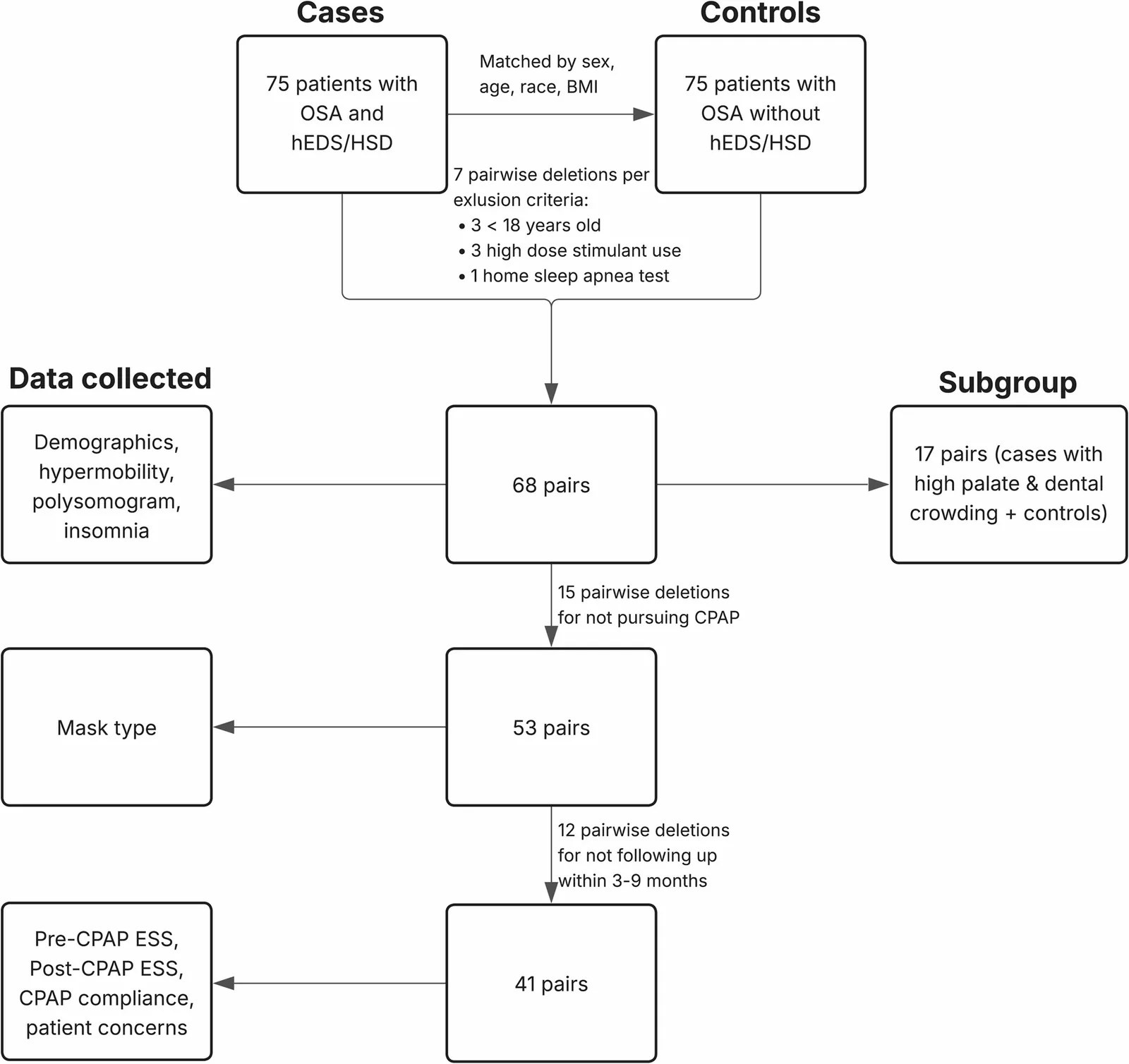
– Flow diagram of enrollment of cases and controls, pairwise deletion, and data collection across study pairs Caption: OSA – obstructive sleep apnea, hEDS – hypermobile Ehlers-Danlos Syndrome, HSD – hypermobility spectrum disorder, CPAP – continuous positive air pressure, ESS – Epworth Sleepiness Scale

75 patients with OSA and hEDS/HSD were identified; 68 met criteria as cases (47% hEDS, 53% HSD, Beighton score 5.71 **±** 2.29) (Table 1). The high prevalence of females (87%) with predominantly European ancestry (94%) is consistent with the hypermobile population observed in clinic [15]. Exact age matching was achieved (34.07 **±** 13.28). Nearest neighbor matching was successful for BMI between cases (32.88 **±** 8.47) and controls (33.07 **±** 8.33). All 68 pairs completed in-clinic PSG after initial sleep clinic consults. 53 pairs pursued CPAP treatment. 41 pairs returned to clinic within 3–9 months of initiating CPAP for reevaluation (Fig. 1). Cases and controls had similar CPAP initiation rates (88% vs. 85%) and 3–9 month follow-up rates (76% vs. 72%). Reasons for loss to follow-up included inability to tolerate CPAP therapy, insurance or equipment barriers, or scheduling difficulties. Baseline characteristics (age, BMI, AHI, and baseline ESS) did not differ meaningfully between patients who returned for follow-up vs. not, suggesting that attrition was unlikely to be systematically related to baseline OSA severity or subjective sleepiness.

**Table 1:** – Cases and controls characteristics

Sex	Cases (68)	Controls (68)	*p*-value
	9 (13%) male, 59 (87%) female		
Race	64 (94%) White, 3 (4%) Black, 1 (1%) Asian		
Age (years)	34.07 **±** 13.28 (range 19–72)	34.07 **±** 13.28 (range 19–72)	1
BMI (kg/m^2^)	32.88 **±** 8.47 (range 18.8–59.6)	33.07 **±** 8.33 (range 19.16–58.93)	0.8934
Insomnia	30 (44.7%)	23 (34.3%)	0.0348
Hypermobility Diagnosis	32 (47%) hEDS, 36 (53%) HDS	N/A	

Caption: *BMI* body mass index, *hEDS* hypermobile Ehlers-Danlos Syndrome, *HSD* hypermobility spectrum disorder

### PSG and clinical outcomes

Insomnia was more prevalent in cases than controls (44.7% vs. 34.3%, *p* < 0.05); sleep efficiency was significantly lower in cases than controls (77.3%±16.4% vs. 82.6%±10.2%, *p* < 0.05). Overall, OSA severity metrics from PSG were similar between cases and controls: AHI (7.2 [13.6] vs. 7.0 [12.7], *p* > 0.05), lowest SpO_2_ (86.0%**±**4.3% vs. 85.6%**±**3.5%, *p* > 0.05), and REM sleep percentage (14.9%±8.1% vs. 12.5%±9.2%, *p* > 0.05) (Table 2). A quarter of the hypermobile cohort (17/68) presented with dental crowding and high-arched palate; no significant differences were found in AHI (7.2 [12.55] vs. 4.9 [11.17], *p* > 0.05) or lowest SpO2 (86.0%**±**6.5% vs. 85.0%**±**8.1%, *p* > 0.05) in this subgroup. Spearman’s rho between Beighton score and AHI is -0.22 and not statistically significant (*p* = 0.076).

**Table 2:** – PSG data comparison

Total sleep time (mins)	Cases (mean ± SE)	Controls (mean ± SE)	*p*-value
	341.81 ± 62.85	326.69 ± 90.66	0.2352
Sleep efficiency	77.3%±16.4%	82.6%±10.2%	0.0396
AHI (#/hour)	7.2 [13.6]	7.2 [12.7]	0.8453
Lowest SpO2	86.0%**±**4.3%	85.6%**±**3.5%	0.5097
REM sleep percentage	14.9%±8.1%	12.5%±9.2%	0.2428
CPAP compliance	75.6%±25.2%	74.6%±26.7%	0.8570
Residual AHI (#/hour)	1.55 [1.75]	1.2 [1.35]	0.1940

Caption: *PSG* polysomnogram, *AHI* apnea-hypopnea index, *SpO2* oxygen saturation, *REM* rapid eye movement, *CPAP* continuous positive air pressure

### Daytime sleepiness outcomes

**Fig. 2:**
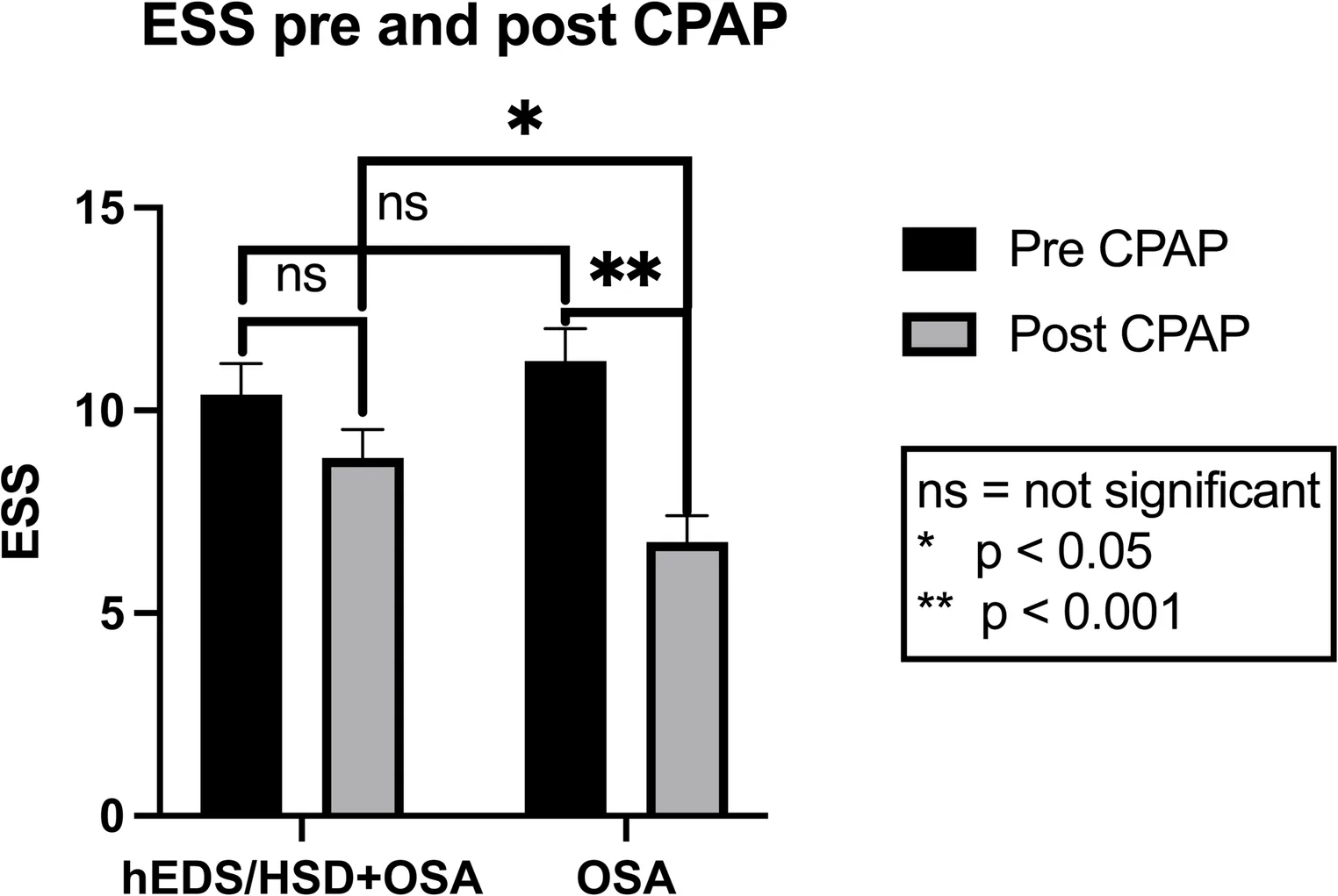
– Epworth Sleepiness Scales before and after CPAP (mean ± standard error of mean) Caption: OSA – obstructive sleep apnea, hEDS – hypermobile Ehlers-Danlos Syndrome, HSD – hypermobility spectrum disorder, CPAP – continuous positive air pressure, ESS – Epworth Sleepiness Scale

Pre-treatment ESS were similar between cases and controls (10.39 ± 4.89 vs. 11.22 ± 5.12, *p* = 0.4490). CPAP significantly reduced ESS in controls by 4.46 (*p* < 0.0001), whereas in cases it produced a smaller, non-significant reduction that trended toward improvement (-1.56, *p* = 0.0620). CPAP treatment was associated with a greater reduction in ESS among controls compared with cases (− 4.46 vs. − 1.56), corresponding to a difference-in-differences of − 2.90 (95% CI − 4.93 to − 0.87, *p* = 0.006), consistent with a significant change over time between groups. After treatment, ESS was higher in cases than controls (8.83 ± 4.47 vs. 6.76 ± 4.15, *p* = 0.0414) (Fig. 2). CPAP compliance, defined as > 4 h per night, were similar between cases and controls (75.6%±25.2% vs. 74.6%±26.7%, *p* = 0.8570). Residual AHI < 5 was achieved with CPAP and similar in both cases and controls (1.55 [1.75] vs. 1.2 [1.35], *p* > 0.05), indicating effective control of apneic events despite differences in symptom improvement.

### CPAP outcomes

**Fig. 3:**
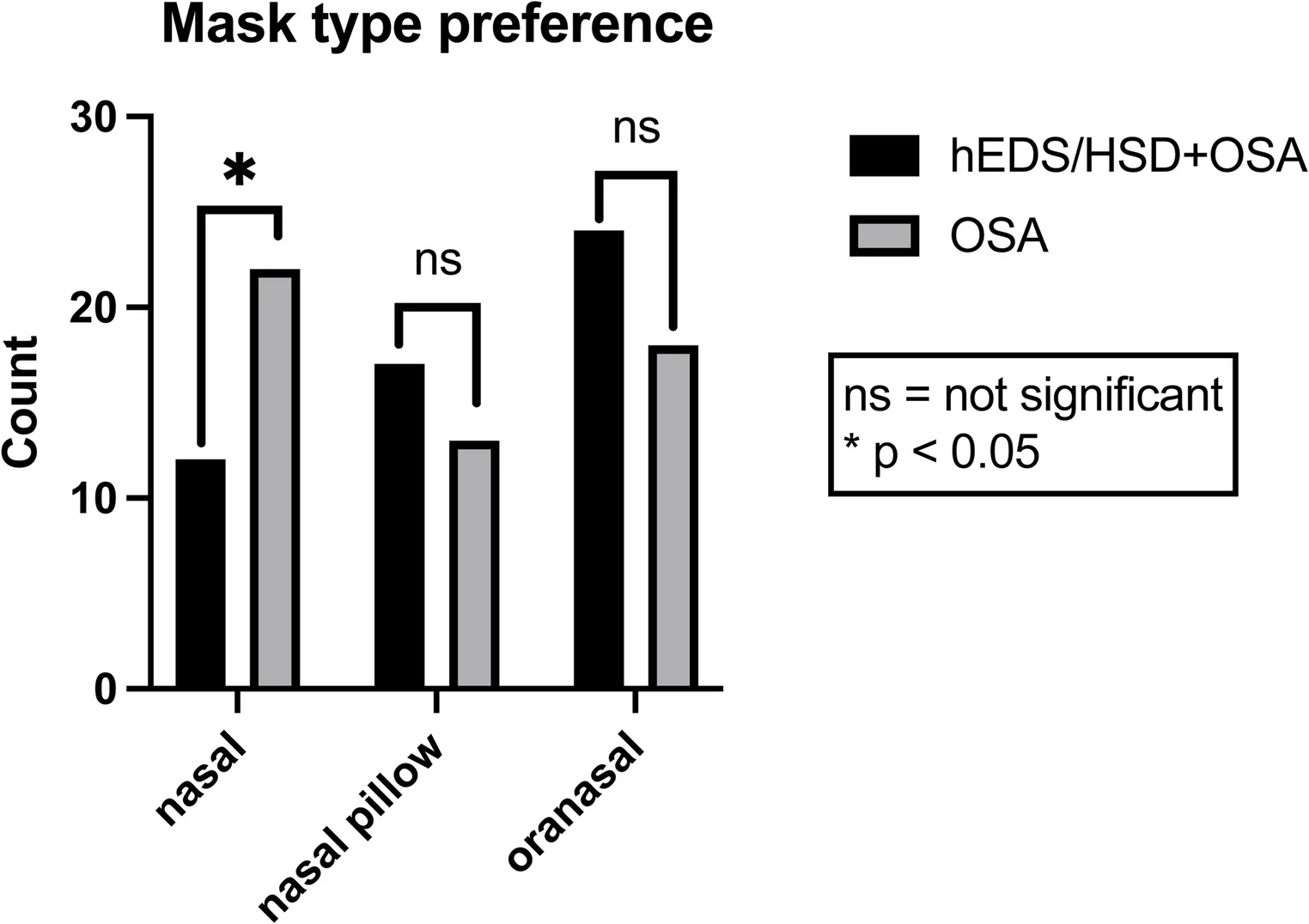
– Distribution of mask selected by cases and controls Caption: OSA – obstructive sleep apnea, hEDS – hypermobile Ehlers-Danlos Syndrome, HSD – hypermobility spectrum disorder

Of the 53 pairs who pursued CPAP, differences in mask preference were observed. Cases were significantly less likely to select nasal masks than controls (22.6% vs. 41.5%, *p* = 0.0184). There was no significant difference in usage of nasal pillow masks (32.1% vs. 24.5%, *p* = 0.3937) and oronasal masks (45.3% vs. 34.0%, *p* = 0.5485) (Fig. 3). At 3–9 months follow-up, cases were more likely to mention concerns about CPAP effect on the nasal mucosa. While both cohorts mentioned nasal/mouth dryness at similar rates (14/41 of cases, 16/41 of controls), only hypermobile patients mentioned more serious concerns such as nosebleeds (3/41) and rawness/ blisters in the nose (1/41).

## Discussion

### OSA severity

Although patients with hEDS and HSD have a higher prevalence of OSA [4], it is unclear whether their connective tissue laxity leads to more severe disease. In this study, hypermobile patients did not demonstrate worse PSG measures of OSA including AHI, lowest oxygen desaturation, or REM sleep percentage when matched to controls on BMI and other demographics. This suggests that while soft-tissue abnormalities in hEDS/HSD may lower the threshold for airway collapsibility and subsequently increase the likelihood of developing OSA, these factors do not intensify OSA severity once apnea is already present. It is possible that once a physiologic threshold for upper-airway collapse is crossed, additional laxity in pharyngeal soft tissues may negligibly worsen obstruction compared to dominant determinants such as adipose load, airway anatomy, and tongue volume [16]. The lack of correlation between Beighton score and AHI in our cohort reinforces this interpretation. These findings suggest that connective tissue abnormalities in hypermobile patients increase OSA risk but do not worsen severity when BMI is equivalent.

### Daytime sleepiness and CPAP response

Cases demonstrated no significant improvements in daytime sleepiness following CPAP therapy compared with matched controls, despite similar adherence and comparable reductions in respiratory disturbance. Our findings were strengthened by direct between-group comparison of ESS change over time, which demonstrated significantly less improvement among cases compared with controls following CPAP treatment. This suggests that patients with hypermobility experienced attenuated symptomatic benefit from CPAP relative to controls. Although the magnitude of improvement was smaller and not statistically significant in cases, ESS still improved after treatment. While this can indicate a diminished effect due to hypermobility, it can also suggest insufficient power to detect a modest benefit as the study was not powered to definitively exclude clinically meaningful benefit within the hypermobile cohort. Therefore, these findings should be interpreted as evidence of comparatively reduced response rather than absence of CPAP efficacy in hypermobile patients.

Residual AHI was similar between cases and controls, indicating hypermobility status does not impair the mechanical effect of CPAP. Therefore, the lack of symptomatic improvement in hypermobile patients cannot be attributed to inadequate treatment of OSA. The residual elevated daytime sleepiness in hypermobile patients may be explained by their concurrent higher rates of insomnia and lower sleep efficiency. Our findings are consistent with literature: hEDS patients have shorter sleep duration, more sleep disturbances, increased sleep latency, and more frequent use of prescription sleep aids according to the largest survey (*n* = 2365) conducted in hEDS population regarding sleep [17]. These findings suggest that in hypermobile patients, excessive daytime sleepiness may not be driven primarily by OSA and have additional contributing mechanisms. We propose several factors here:

1. Chronic widespread pain or pain in > 2 limbs lasting > 3 months is a diagnostic criterion for hEDS/HSD in the 2017 diagnostic framework [1]. In EDS patients, chronic pain can fragment sleep, increase nocturnal awakenings, and impair sleep initiation and maintenance [18]. On the other hand, shorter sleep duration has been linked to higher prevalence of chronic musculoskeletal pain [19]. This bidirectional impact of pain and sleep may be particularly problematic for hEDS/HSD patients as it creates a vicious self-exacerbating cycle.
2. The increased prevalence of dysautonomia, particularly postural orthostatic tachycardia syndrome (POTS) and mast cell activation disorder (MCAS), in hEDS/HSD is theorized to be a result of increased vascular laxity from defective collagen synthesis [20]. While enhanced sympathetic drive and dysautonomia are generally linked to nonrestorative sleep and sleep disorders [21], direct evidence characterizing this relationship in this specific patient population is limited. It is worth noting however that severe autonomic symptoms in hypermobile populations is associated with poor quality of life due to chronic pain and fatigue [22].
3. Psychiatric disorders, especially anxiety and depression, are highly prevalent in hEDS/HSD population; psychological processes such as fear, emotional distress, and arousal are also heightened in hEDS/HSD [23]. Heightened physiologic arousal driven by anxiety and hypervigilance can make it more difficult for patients to relax at bedtime, potentially worsening sleep onset and maintenance. This increased arousal may also contribute to discomfort or intolerance with CPAP therapy, thereby reducing its perceived benefit for OSA.

Clinically, successful treatment of daytime sleepiness in hypermobile patients may require addressing all these comorbidities in parallel to OSA. When these factors remain uncorrected, they may mask the symptomatic benefit derived from CPAP. Thus, the persistent daytime sleepiness observed in hypermobile patients likely reflects a multifactorial disorder of sleep regulation, in which OSA is only one component rather than the dominant driver. It is worth noting that symptoms frequently reported in hEDS/HSD due to these comorbidities only contribute partially to hypersomnolence, and the full symptom burden cannot be captured by ESS alone, which specifically measures subjective daytime sleepiness rather than fatigue, exhaustion, or broader functional impairment.

The relatively mild baseline OSA severity in both cohorts (16.8 ± 28.0 vs. 12.4 ± 16.3) may have constrained the magnitude of symptom improvement with CPAP, particularly in the hypermobile group where non-OSA contributors to sleepiness are prevalent. This raises the possibility of a floor effect, where limited physiologic burden from OSA restricts the potential for measurable improvement in ESS despite effective treatment. Although stratified analyses by OSA severity (mild, moderate, severe) would be informative, the current sample size limited meaningful subgroup comparisons. Nonetheless, the significant difference of post-CPAP ESS between cohorts supports the hypothesis of residual sleepiness not driven primarily by OSA.

### Craniofacial

Craniofacial factors such as maxillary constriction, narrow dental arch, and increased palatal height are well-established risk factors for OSA [24]. Particularly, a high-arched palate is associated with increased upper-airway collapsibility and higher AHI by reducing the transverse dimension of the maxillary arch and narrowing the upper airway [25]. High-arched palate with dental crowding is one of the twelve diagnostic systemic manifestations of hEDS [1], raising the expectation that hypermobile patients would exhibit more severe disease. However, our subgroup of hypermobile patients with high-arched palate and dental crowding (25% of cases) did not demonstrate worse apnea severity than matched controls. One plausible explanation is that our control group, by virtue of already having clinically significant OSA, may share similar underlying predispositions for craniofacial abnormalities. In this context, the absence of higher AHI in hypermobile patients may reflect that the two groups have similar prevalence of structural airway risk factors for OSA, rather than a lack of impact of craniofacial abnormalities on OSA severity. The lack of statistical significance may also be attributable to insufficient power in this exploratory subgroup analysis.

### Mucosal effects

Hypermobile patients more frequently reported CPAP-related nasal mucosal adverse effects (epistaxis and blisters) despite similar rates of reporting nonspecific dryness. hEDS patients are more likely to bleed due to capillary fragility and connective tissue abnormalities [26], as measured by validated bleeding assessment tools such as the International Society on Thrombosis and Haemostasis Bleeding Assessment Tool (ISTH-BAT) [27]. Mucosal bleeding such as epistaxis causes more quality-of-life distress in hEDS/HSD patients, as revealed by qualitative interviewing [28]. The distribution of airflow within the nasal cavity is highly dependent on individual anatomy, but most airflow typically traverses the common nasal meatus with greater shear stress on the mucosa [29]. Nasal mucosal effects are likely multifactorial, influenced by mask type and fit, airflow patterns, mouth leaks, use of humidification, and concomitant medications such as anticoagulants. Larger, controlled studies are warranted to better understand the contributors to nasal mucosal adverse effects in this population. Regardless, the differential mask preferences observed in this study highlight the importance of individualized mask selection and counseling in patients with hEDS/HSD.

Granular CPAP metrics including mean nightly usage, mask leak, pressure distributions, and humidification settings were not analyzed. These parameters may influence comfort, mucosal irritation, and perceived benefit without affecting adherence classification or residual AHI. This limitation is particularly relevant given the increased mucosal complaints reported by hypermobile patients and should be considered in future studies when investigating subjective sleepiness outcomes.

### Strengths and limitations

This study has several strengths. Cases and controls were matched on key demographic factors, enabling isolation of hypermobility status as the primary variable of interest. Standardized, laboratory-based PSG and structured follow-up visits at our single institution strengthened the consistency of outcome evaluation. The inclusion of qualitative patient-reported experiences also provided clinically relevant context not typically captured in retrospective analyses. Both groups achieved higher-than-average adherence of around 75%, allowing daytime sleepiness to be evaluated without confounding from noncompliance.

This study has several limitations inherent to its retrospective design. The small sample size of 68 pairs may limit the ability to detect smaller differences, especially in subgroups. We did not perform a formal power calculation due to the exploratory nature of this study. Missing data may introduce additional bias. ESS provides a validated measure of daytime sleepiness but cannot identify the specific contributors in each patient such as pain, psychiatric factors, or other symptoms. Severity of hypermobility was approximated by Beighton score, which remains limited in capturing the full spectrum of systemic connective tissue involvement and may not correlate with airway tissue laxity, dysautonomia or pain severity [14]. Findings should also be interpreted with caution in terms of generalizability, as data were obtained from a single institution and may not represent broader patient demographics.

An important limitation of this study is the lack of systematic assessment and.

adjustment for mentioned common comorbidities of hEDS/HSD as key potential confounders, including chronic pain severity, psychiatric comorbidities, and the use of medications such as antidepressants that may influence subjective sleepiness. As these factors may differ between patients with hEDS/HSD and controls, future studies with better-matched cohorts and more comprehensive covariate measurement are needed to clarify their contribution to observed differences in CPAP response.

### Conclusion

While hEDS/HSD has been identified as risk factors for OSA, these diagnoses do not exacerbate OSA severity. Hypermobile patients with OSA demonstrated less improvement in daytime sleepiness after CPAP therapy despite similar disease severity and treatment adherence. These results suggest that OSA represents only one contributor to daytime fatigue in hypermobile patients and that a multidisciplinary approach including evaluation of autonomic dysfunction, insomnia, psychiatric comorbidities, and chronic pain may be necessary to optimize outcomes. Mucosal fragility in hypermobile patients warrants further investigation using granular CPAP data such as mask leak, humidification, and pressure distribution to inform mask selection and treatment optimization. 

## Data Availability

Data available on reasonable request from the corresponding author.

## Abbreviations

AHI: apnea–hypopnea index
APAP: auto–titrating positive airway pressure
BMI: body mass index
COPD: chronic obstructive pulmonary disease
CPAP: continuous positive airway pressure
ESS: Epworth Sleepiness Scale
hEDS: hypermobile Ehlers–Danlos syndromes
HSD: hypermobile spectrum disorder
ISTH: BAT–International Society on Thrombosis and Haemostasis Bleeding Assessment Tool
OSA: obstructive sleep apnea
PAP: positive airway pressure
PSG: polysomnogram
RDI: respiratory disturbance index
REM: rapid eye movement

## References

[1] Malfait F, Francomano C, Byers P et al (2017) The 2017 international classification of the Ehlers-Danlos syndromes. Am J Med Genet C Semin Med Genet 175:8–26. 10.1002/ajmg.c.31552 28306229 10.1002/ajmg.c.31552

[2] Aubry-Rozier B, Schwitzguebel A, Valerio F et al (2021) Are patients with hypermobile Ehlers-Danlos syndrome or hypermobility spectrum disorder so different? Rheumatol Int 41:1785–1794. 10.1007/s00296-021-04968-3 34398260 10.1007/s00296-021-04968-3 PMC8390400

[3] Gaisl T, Giunta C, Bratton DJ et al (2017) Obstructive sleep apnoea and quality of life in Ehlers-Danlos syndrome: A parallel cohort study. Thorax 72:729–735. 10.1136/thoraxjnl-2016-209560 28073822 10.1136/thoraxjnl-2016-209560

[4] Sedky K, Gaisl T, Bennett DS (2019) Prevalence of Obstructive Sleep Apnea in Joint Hypermobility Syndrome: A Systematic Review and Meta-Analysis. J Clin Sleep Med 15:293–299. 10.5664/jcsm.7636 30736885 10.5664/jcsm.7636 PMC6374081

[5] Guilleminault C, Primeau M, Chiu H-Y et al (2013) Sleep-disordered breathing in Ehlers-Danlos syndrome: a genetic model of OSA. Chest 144:1503–1511. 10.1378/chest.13-0174 23929538 10.1378/chest.13-0174

[6] Menton SM, Fairweather D, Bruno KA et al (2024) Laryngological Complaint Prevalence in Hypermobile Ehlers-Danlos or Hypermobility Spectrum Disorders. Laryngoscope 134:773–778. 10.1002/lary.30964 37597175 10.1002/lary.30964 PMC10841389

[7] Antunes D, Nicot R, Bovis M, Ferri J (2025) Oral and maxillofacial clinical features of Ehlers-Danlos syndrome: a systematic review. Oral Surg Oral Med Oral Pathol Oral Radiol 140:594–615. 10.1016/j.oooo.2025.05.010 40615270 10.1016/j.oooo.2025.05.010

[8] Hertel AK, Jones JT, Lytch A et al (2025) Prevalence of psychiatric and sleep disorders and their impact on quality of life in children with hypermobile Ehlers-Danlos syndrome: an observational study. Rheumatol Int 45:81. 10.1007/s00296-025-05836-0 40131551 10.1007/s00296-025-05836-0 PMC11937059

[9] Voermans NC, Knoop H, van de Kamp N et al (2010) Fatigue is a frequent and clinically relevant problem in Ehlers-Danlos Syndrome. Semin Arthritis Rheum 40:267–274. 10.1016/j.semarthrit.2009.08.003 19878973 10.1016/j.semarthrit.2009.08.003

[10] Ayas FY, Özcebe LH (2025) The relationship between fatigue, sleep quality, and sleep deprivation. Sleep Breath 29:73. 10.1007/s11325-024-03231-w 39804542 10.1007/s11325-024-03231-w

[11] Emsellem HA, Colwell HH, Cronin J et al (2025) Fatigue is distinct from sleepiness and negatively impacts individuals living with obstructive sleep apnea (OSA): results from qualitative research of individuals with OSA. Health Qual Life Outcomes 23:26. 10.1186/s12955-025-02355-1 40128860 10.1186/s12955-025-02355-1 PMC11934705

[12] Harris PA, Taylor R, Thielke R et al (2009) Research electronic data capture (REDCap)--a metadata-driven methodology and workflow process for providing translational research informatics support. J Biomed Inf 42:377–381. 10.1016/j.jbi.2008.08.010 10.1016/j.jbi.2008.08.010 PMC2700030 18929686

[13] Johns MW (1991) A New Method for Measuring Daytime Sleepiness: The Epworth Sleepiness Scale. Sleep 14:540–545. 10.1093/sleep/14.6.540 1798888 10.1093/sleep/14.6.540

[14] Malek S, Reinhold EJ, Pearce GS (2021) The Beighton Score as a measure of generalised joint hypermobility. Rheumatol Int 41:1707–1716. 10.1007/s00296-021-04832-4 33738549 10.1007/s00296-021-04832-4 PMC8390395

[15] Castori M, Camerota F, Celletti C et al (2010) Ehlers–Danlos syndrome hypermobility type and the excess of affected females: Possible mechanisms and perspectives. Am J Med Genet Pt A 152A:2406–2408. 10.1002/ajmg.a.33585 10.1002/ajmg.a.33585 20684008

[16] Hartfield PJ, Janczy J, Sharma A et al (2023) Anatomical determinants of upper airway collapsibility in obstructive sleep apnea: A systematic review and meta-analysis. Sleep Med Rev 68:101741. 10.1016/j.smrv.2022.101741 36634409 10.1016/j.smrv.2022.101741 PMC11493082

[17] Crews-Stowe C, Tudini F, Jung M-K et al (2025) Sleep Characteristics in Individuals with Ehlers-Danlos Syndrome. Med Sci (Basel) 13:85. 10.3390/medsci13030085 40700114 10.3390/medsci13030085 PMC12286137

[18] Voermans NC, Knoop H, Bleijenberg G, van Engelen BG (2010) Pain in ehlers-danlos syndrome is common, severe, and associated with functional impairment. J Pain Symptom Manage 40:370–378. 10.1016/j.jpainsymman.2009.12.026 20579833 10.1016/j.jpainsymman.2009.12.026

[19] Li C, Huang H, Xia Q, Zhang L (2024) Association between sleep duration and chronic musculoskeletal pain in US adults: a cross-sectional study. Front Med (Lausanne) 11:1461785. 10.3389/fmed.2024.1461785 39386748 10.3389/fmed.2024.1461785 PMC11461308

[20] Aziz Q, Harris LA, Goodman BP et al (2025) AGA Clinical Practice Update on GI Manifestations and Autonomic or Immune Dysfunction in Hypermobile Ehlers-Danlos Syndrome: Expert Review. Clin Gastroenterol Hepatol 23:1291–1302. 10.1016/j.cgh.2025.02.015 40387691 10.1016/j.cgh.2025.02.015

[21] Olivares MJ, Toledo C, Ortolani D et al (2022) Sleep dysregulation in sympathetic-mediated diseases: implications for disease progression. Sleep 45:zsac166. 10.1093/sleep/zsac166 35878762 10.1093/sleep/zsac166

[22] Collins Hutchinson ML, Liang E, Fuster E, Blitshteyn S (2025) Autonomic symptom burden, comorbidities and quality of life in women with Hypermobility Spectrum Disorders and hypermobile Ehlers-Danlos syndrome. Auton Neurosci 262:103356. 10.1016/j.autneu.2025.103356 41118678 10.1016/j.autneu.2025.103356

[23] Bulbena-Cabré A, Baeza-Velasco C, Rosado-Figuerola S, Bulbena A (2021) Updates on the psychological and psychiatric aspects of the Ehlers-Danlos syndromes and hypermobility spectrum disorders. Am J Med Genet C Semin Med Genet 187:482–490. 10.1002/ajmg.c.31955 34806831 10.1002/ajmg.c.31955

[24] Neelapu BC, Kharbanda OP, Sardana HK et al (2017) Craniofacial and upper airway morphology in adult obstructive sleep apnea patients: A systematic review and meta-analysis of cephalometric studies. Sleep Med Rev 31:79–90. 10.1016/j.smrv.2016.01.007 27039222 10.1016/j.smrv.2016.01.007

[25] Mahony D, Harding S, Chowdhury CR et al (2025) Correlation Between Severity of Obstructive Sleep Apnea and Dental Arch Form in Adults. J Clin Med 14:7183. 10.3390/jcm14207183 41156050 10.3390/jcm14207183 PMC12565247

[26] Jesudas R, Chaudhury A, Laukaitis CM (2019) An update on the new classification of Ehlers-Danlos syndrome and review of the causes of bleeding in this population. Haemophilia 25:558–566. 10.1111/hae.13800 31329366 10.1111/hae.13800

[27] Kumskova M, Flora GD, Staber J et al (2023) Characterization of bleeding symptoms in Ehlers-Danlos syndrome. J Thromb Haemost 21:1824–1830. 10.1016/j.jtha.2023.04.004 37179130 10.1016/j.jtha.2023.04.004

[28] Jesudas R, Mathena SA, Zhang KL et al (2025) Distressing Bleeding Symptoms in Interviews of Patients With Hypermobile Ehlers–Danlos Syndrome and Hypermobility Spectrum Disorder. 10.1111/hae.70174. Haemophilia hae.70174 10.1111/hae.70174 41358659

[29] Yu S, Liu Y, Sun X, Li S (2008) Influence of nasal structure on the distribution of airflow in nasal cavity. Rhinology 46:137–143 18575016

